# The impact of emotional intelligence on life satisfaction among Chinese nurses: A chain mediating model

**DOI:** 10.3389/fpsyg.2023.1125465

**Published:** 2023-02-17

**Authors:** Yuan Qin, Jiao Liu, Dongmei Wu

**Affiliations:** ^1^School of Nursing, Chengdu Medical College, Chengdu, China; ^2^Chongqing Mental Health Center, Chongqing, China; ^3^School of Nursing, Zunyi Medical University, Zunyi, China; ^4^Department of Nursing, The Clinical Hospital of Chengdu Brain Science Institute, MOE Key Laboratory for Neuroinformation, University of Electronic Science and Technology of China, Chengdu, China

**Keywords:** emotional intelligence, self-efficacy, resilience, life satisfaction, China, nurses

## Abstract

**Introduction:**

Nurses’ life satisfaction exerts a positive impact on their professional careers, and it seriously affects their physical and mental health. Low life satisfaction has become a key factor in the global shortage of nurses. Emotional intelligence may protect nurses from negative emotions that can affect the care they provide, as well as their life satisfaction. In this study, we aims to explore the impact of emotional intelligence on life satisfaction, and even verify the chain mediating effect of self-efficacy and resilience on this relationship among Chineses nurses.

**Method:**

The Emotional Intelligence Scale, the General Self, Efficacy Scale, the Connor-Davidson Resilience Scale, and the Satisfaction with Life Scale were used to survey 709 nurses in southwest China. To analyze mediating effects, SPSS 26.0 and Process V3.3 were used for statistical processing.

**Result:**

Emotional intelligence positively predicted life satisfaction. Meanwhile, it was also found that emotional intelligence and life satisfaction were continuously mediated by self-efficacy and resilience, and the indirect effect value was 0.033, accounting for 17.37%.

**Conclusion:**

This study reveals how emotional intelligence affects nurses’ life satisfaction. The results of this study have certain implications for nurses to better balance their career and life. Nursing managers should provide nurses with a favorable working environment from the perspective of positive psychology, improve their sense of self-efficacy and resilience, ultimately improve their life satisfaction.

## Introduction

Nursing is a special profession closely related to people’s health and life. Many nurses are facing a series of stressful situations, such as emotional exhaustion, low self-efficacy, low personal achievement, and low life satisfaction (Molero Jurado et al., 2019). Life satisfaction refers to the degree of subjective satisfaction generated by individual needs and wishes in various aspects (Diener, 1985). A recent study of nursing staff demonstrated that nurses who were dissatisfied with their lives were more likely to suffer from psychological disorders, with a risk as high as 2.4 times (Zakeri et al., 2021). Numerous studies have discovered that nurses’ life satisfaction is associated with resilience, gratitude disposition, job satisfaction, compassion, and self-efficacy (Yu and Lee, 2018; Zakeri et al., 2021; Milutinović et al., 2022). Furthermore, life satisfaction is positively correlated with occupational burnout, and nurses with low life satisfaction are prone to occupational burnout (Zborowska et al., 2021). Therefore, improving the life satisfaction of nurses is very important for them to maintain their careers and promote mental health.

Emotional intelligence refers to a series of ability that helps individuals accurately evaluate and understand the emotional needs of themselves and others, and rationally use emotional information to promote cognitive activities (Salovey and Mayer, 1990). A study on student registered nurse anesthetists found that emotional intelligence can help nursing students reduce the impact of negative emotions on important decisions, and positively impacts the care they provide (Christianson et al., 2021). Meanwhile, a study on Spanish nurses indicated that emotional intelligence can reduce nurses’ burnout and psychosomatic complaining, while protecting them from the negative effects of psychosocial risks and positively affecting their job satisfaction (Soto-Rubio et al., 2020). Similarly, a prior study confirmed that emotional intelligence directly predicts nurses’ life satisfaction, which means that nurses with high emotional intelligence may be more satisfied with their lives (Montes-Berges and Augusto-Landa, 2014). Thus, the hypothesis is proposed:

*Hypothesis 1*: Emotional intelligence significantly predicts life satisfaction.

Self-efficacy is the belief that a person can accomplish a task or achieve a goal (Deer et al., 2018), which is considered a judgment made by nursing students about their ability to handle various tasks (Arribas-Marín et al., 2021). The factor affects nurses’ motivation, actions, and behaviors during patient care (Cziraki et al., 2018). During the COVID-19 pandemic, self-efficacy was also considered beneficial to Chinese frontline nurses’ psychological health, which can effectively reduce their anxiety and depression (Hu et al., 2020). Moreover, research on medical professionals demonstrated that emotional intelligence is positively associated with self-efficacy; that is, the stronger the emotional management ability, the stronger the self-efficacy (Zeb et al., 2021). Furthermore, researchers also found that nursing students’ self-efficacy directly affects life satisfaction (Arribas-Marín et al., 2021). When nurses have a high sense of self-efficacy, they work efficiently in demanding situations, maintain good stress management skills, and ultimately improve life satisfaction. Therefore, we propose the assumption:

*Hypothesis 2*: Self-efficacy might mediate the relationship between emotional intelligence and life satisfaction.

Resilience means an individual capacity to adapt to adversity, trauma, and other major stressors (Aburn et al., 2016). Nurses’ resilience has been considered a skill that can help nurses overcome difficulties in the workplace (Young and Rushton, 2017), which contributes to improving healthcare quality and ensuring the sustainability of the healthcare system (Epstein and Krasner, 2013). Meanwhile, resilience has been thought to be positively correlated with emotional intelligence among critical care nurses (Lampreia-Raposo et al., 2022). Furthermore, a randomized controlled trial demonstrated that nurses’ resilience can be improved by emotional intelligence training (Mao et al., 2021). In addition, high levels of nurses’ resilience can act as a protective factor against adverse outcomes, such as burnout, anxiety, and depression (Baskin and Bartlett, 2021), it also can improve nurses’ job satisfaction (Brown et al., 2018), quality of life (Alhawatmeh et al., 2021), general well-being (García-Izquierdo et al., 2018), and life satisfaction (Labrague, 2021). Therefore, We hypothesize that:

*Hypothesis 3*: The relationship between emotional intelligence and life satisfaction can be mediated by resilience.

Numerous studies have depicted that emotional intelligence, self-efficacy, and resilience are positively associated with job satisfaction, well-being, and life satisfaction among nurses (Yu et al., 2019; Li et al., 2021; Platania et al., 2022). Researchers pointed out that self-efficacy directly predicts resilience in Chinese nurses, which assists them in dealing with changes and improving problem-solving ability (Ren et al., 2018). High levels of emotional intelligence endow nurses with a positive attitude toward frustration and increase their belief in coping with difficulties, which is also conducive to adapting to pressure, accelerating recovery from adversity, improving job satisfaction and happiness, and ultimately improving life satisfaction. Consequently, we pose the assumption:

*Hypothesis 4*: Self-efficacy and resilience jointly mediate the relationship between emotional intelligence and life satisfaction.

In conclusion, while previous literatures have separately investigated the relationship between emotional intelligence, self-efficacy, resilience, and life satisfaction, few studies have depicted how emotional intelligence affects life satisfaction through self-efficacy and resilience. Thus, this study aims to explore the potential internal mechanism between these four variables, and even verify the intermediary role of self-efficacy and resilience.

## Materials and methods

### Participants

In this study, participants from two provinces in Southwest China (Sichuan and Yunnan) were recruited through online advertising. Participants were required to obtain nursing certificate and be able to complete the questionnaire independently through the Internet. At the same time, participants need to have worked in the nursing field for at least 1 year, and then, they also could not be mentally ill or dependent on drugs or alcohol. Before the study, all participants were fully informed and participated in this study on a voluntary basis. Based on this, they provided written informed consent during the study. In total, 756 nurses completed the survey, of which 709 were deemed valid, and the effective response rate was 93.7%. Table 1 shows the participants’ basic characteristics.

**Table 1:** Social demographic features and differences of nurses’ life satisfaction scores.

Variables	Frequency	Life Satisfaction	*t/F*	*p*
	(percentage)	(M ± SD)		
Gender			−1.433	0.152
Male	66(9.3%)	20.89 ± 5.95		
Female	643(90.7%)	21.91 ± 5.45		
Age(year)			4.744	0.009
≤30	379(53.46%)	21.23 ± 5.26		
31 ~ 39	215(30.32%)	22.50 ± 5.48		
≥40	115(16.22%)	22.50 ± 6.12		
Educational level			3.361	0.018
Technical secondary school	37(5.22%)	19.22 ± 5.88		
Junior college	270(38.08%)	21.99 ± 5.34		
Undergraduate	398(56.14%)	21.91 ± 5.54		
Postgraduate	4(0.56%)	25.00 ± 4.76		
Working years			−3.176	0.002
<10	474(66.85%)	21.36 ± 5.26		
≥10	235(33.15%)	22.74 ± 5.87		
Nursing title			4.659	0.003
None	163(22.99%)	20.67 ± 5.28		
Primary	366(51.62%)	21.81 ± 5.31		
Intermediate	170(23.98%)	22.85 ± 5.90		
Vice senior	10(1.41%)	23.30 ± 6.46		
Marital status			6.818	<0.001
Unmarried	198(27.93%)	20.56 ± 5.30		
Married	480(67.70%)	22.50 ± 5.44		
Divorced	26(3.67%)	19.65 ± 5.49		
Widowed	2(0.28%)	21.50 ± 4.95		
Other	3(0.42%)	15.00 ± 9.17		

### Measures

All measures in this study were carried out in Chinese language.

### Basic sociodemographic data

A self-designed questionnaire was adopted, including gender, age, educational level, working years, nursing title, and marital status.

### Emotional intelligence

The Self-reported Emotional Intelligence Scale (WEIS) was designed and validated by Wong and Law from Hong Kong (Wong and Law, 2002). The scale includes 16 items in 4 subscales, such as “I can usually guess my friends’ emotions from their behavior.” A seven-point scale system was used: “1″ = “not at all” and “7” = “very much.” The higher scores suggesting the better individual’s emotional intelligence. In the sample of China, the WEIS has been proved to be valid and reliable (Cronbach’s α = 0.83; Wong and Law, 2002). The reliability of the 4 components in this study was from 0.81 to 0.84.

### Self-efficacy

The General Self-Efficacy Scale (GSES) was adopted to evaluate an individual self-efficacy. Zhang et al. developed the Chinese version in 1995 (Zhang and Schwarzer, 1995) and it has been widely used by the Chinese population. The GSES is a one-dimensional structure with 10 items in total, and the scale is rated on a 4-point Likert response format, with “1” meaning “definitely false” and “4” meaning “definitely true.” This questionnaire has been measured previously by Wang et al. on Chinese college students with a reported good internal consistency and Cronbach’s α of 0.83 (Wang et al., 2020). For the current sample, Cronbach’s α = 0.879.

### Resilience

The Connor-Davidson Resilience Scale (CD-RISC) is used to evaluate individual resilience, designed by Campbell-Sills and Stein (2007) and translated by Wang et al. (2010). In the sample of China, it is verified as an efficient measurement of resilience (Cronbach’s α = 0.91; Wang et al., 2010). The measurement is a unidimensional dimension consisting of 10 items, using a 5-point Likert scale, with “1” = “none” and “5” = “always.” Sample items were: “When things change, I can adapt,” “The positive side is always on my mind when facing difficulties,” etc. A Cronbach’s coefficient of 0.927 was determined in the current study.

### Life satisfaction

An assessment of participants’ satisfaction with life was conducted using the Satisfaction with Life Scale (SWLS) designed by Diner (Diener, 1985). The scale contains 5 items, and the Chinese version is highly reliable and valid (Xu et al., 2021). Nurses agreed to seven Likert responses from 0 to 7, with “1″ = “never” and “7″ = “always.” Cronbach’s coefficient for the SWLS was 0.8744 in this study, with higher scores indicating greater satisfaction with life.

### Procedure

The researchers recruited all participants and provided online informed content through online platforms. Before the investigation, researchers have received unified training to ensure that all researchers follow the same guidelines, and then the researchers were assigned to each hospital. The questionnaires indicated the purpose of the investigation, the filling method, and commitment to the principle of confidentiality. The approval of the Ethics Committee of Chengdu Fourth Hospital was obtained prior to this study (number ChiCTR1900020715).

### Statistical analysis

SPSS 26.0 and Process V3.3 were used for statistical analysis. An analysis of nurses’ life satisfaction was performed using the samples t-test or one-way analysis of variance. An analysis of Pearson’s correlations was conducted on both factors of emotional intelligence, self-efficacy, resilience, and life satisfaction. Process V3.3 Model 6 was adopted to test the mediation effect. The Bootstrap was used to test the significance of the mediating effects with 5,000 repeated sampling, and Two-sided inspection level α = 0.05.

## Results

Table 1 shows social demographic features and differences of nurses’ life satisfaction scores. Seven hundred and nine nurses met the inclusion criteria, the differences in life satisfaction scores among subgroups with different age, educational level, working years, nursing title, and marital status were statistically significant (*P* < 0.05).

### Control variables

Based on the statistical analysis in Table 1, the control variables were age, educational level, working years, nursing title, and marital status.

### Pearson’s correlation analysis

Statistics and the Pearson’s correlation matrix for each variable are shown in Table 2. The result indicated that nurse’s emotional intelligence was positively correlated with self-efficacy, resilience, and life satisfaction. Self-efficacy was positively associated with resilience and life satisfaction. Meanwhile, nurse’s resilience was also positively associated with life satisfaction.

**Table 2:** The statistical descriptions and associations among study variables.

Variables	*Mean*	*SD*	1	2	3	4
1. Emotional intelligence	83.25	12.85	–			
2. Self-efficacy	37.26	5.55	0.634**	–		
3. Resilience	27.32	3.95	0.701**	0.700**	–	
4. Life satisfaction	21.82	5.51	0.449**	0.487**	0.511**	–

** *P*<0.01.

### The mediation effect analysis

Table 3 reports the mediation effect analysis among variables. After age, educational level, working years, nursing title, and marital status were controlled, emotional intelligence significantly predicted life satisfaction (*β* = 0.057, *p* < 0.01); emotional intelligence significantly predicted self-efficacy (*β* = 0.190*, p* < 0.001); and self-efficacy predicted life satisfaction (*β* = 0.288, *p* < 0.001), meaning that self-efficacy partially mediated between emotional intelligence and life satisfaction. At the same time, emotional intelligence significantly predicted resilience (*β* = 0.174, *p* < 0.001) and resilience predicted life satisfaction (*β* = 0.261, *p* < 0.001), meaning that resilience played a partial intermediary effect between emotional intelligence and life satisfaction. Furthermore, self-efficacy predicted resilience (*β* = 0.662, *p* < 0.001). In a word, self-efficacy and resilience played a continuous intermediary effect between nurses’ emotional intelligence and life satisfaction.

**Table 3:** Regression analysis among study measures.

Variables	*β*	*t*	*P*	LLCI	ULCI	*R^2^*	*F*
**Result variable: Self-efficacy**							
Predictor emotional intelligence	0.190	21.424	<0.001	0.173	0.207	0.424	86.125
**Result variable: Resilience**							
Predictor emotional intelligence	0.174	13.497	<0.001	0.148	0.199	0.631	170.909
Mediator self-efficacy	0.662	15.562	<0.001	0.579	0.746		
**Result variable: Life satisfaction**							
Predictor emotional intelligence	0.057	2.908	0.004	0.019	0.096	0.305	38.313
Mediator 1 Self-efficacy	0.288	4.284	<0.001	0.156	0.420		
Mediator 2 Resilience	0.261	5.072	<0.001	0.160	0.362		
**Result variable: Life satisfaction**							
Independent variable emotional intelligence	0.190	13.160	<0.001	0.162	0.218	0.216	32.267

**Figure 1:**
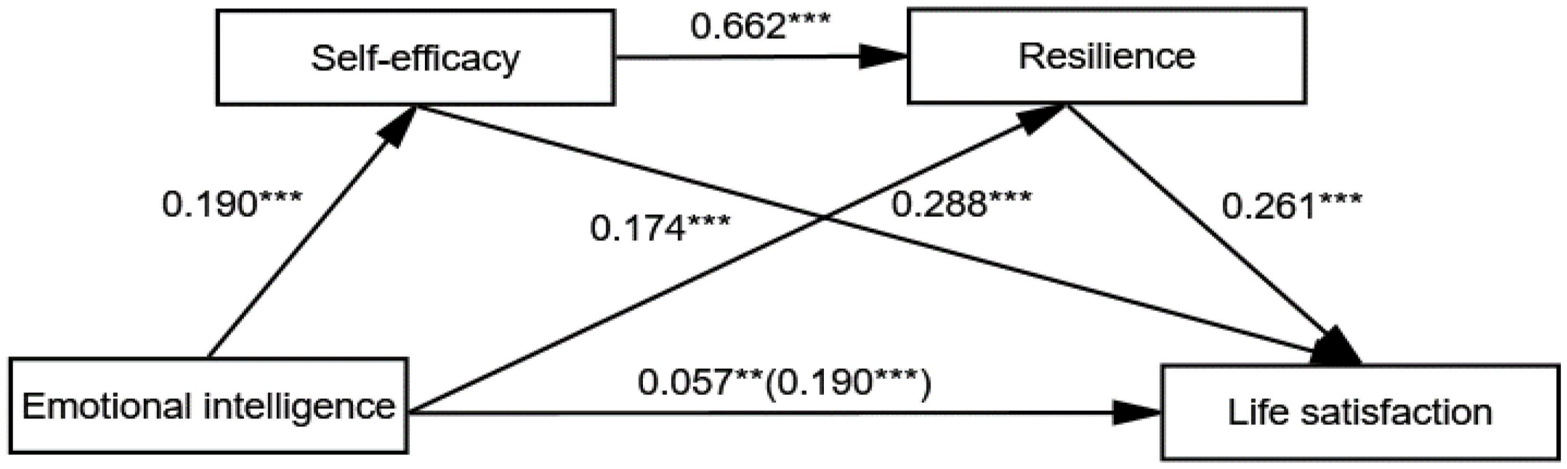
Model diagram of the effect of emotional intelligence on life satisfaction (**p < 0.01, ***p < 0.001).

The continuous intermediary effect of self-efficacy and resilience was verified by the Bootstrap method (5,000 repeated sampling), with a 0.133 indirect effect value [95% CI: 0.100, 0.164], excluding 0 (see Table 4). Especially, the 95% confidence intervals of the three indirect effect paths did not contain 0. First, the effect size of path1 (emotional intelligence → self-efficacy → life satisfaction) was 28.95%, and the intermediary effect (self-efficacy = 0.055, 95% CI = 0.027–0.083) was significant. Second, the path2 (emotional intelligence → resilience → life satisfaction) accounted for 23.68%, and the intermediary effect (resilience = 0.045, 95% CI = 0.024–0.067) was significant. Third, the path3 (emotional intelligence → self-efficacy → resilience → life satisfaction) accounted for 17.37%, and the intermediary effect value was 0.033 [95% CI: 0.017, 0.049]. Figure 1 shows the multiple mediating model.

**Table 4:** Bootstrap analysis of the chain mediating model.

Path	Effect	Boot SE	Boot	Boot	Effect ratio
			LLCI	ULCI	
Total effect	0.190	0.014	0.162	0.218	100%
Direct effect	0.057	0.020	0.019	0.096	30.00%
Total indirect effect	0.133	0.016	0.100	0.164	70.00%
Path1	0.055	0.014	0.027	0.083	28.95%
Path2	0.045	0.011	0.024	0.067	23.68%
Path3	0.033	0.008	0.017	0.049	17.37%
Comparsion1 (Path1 and Path2)	0.009	0.022	−0.035	0.054	
Comparsion2 (Path1 and Path3)	0.022	0.020	−0.017	0.061	
Comparsion3 (Path2 and Path3)	0.012	0.006	−0.001	0.026	

Path1: Emotional intelligence → Self-efficacy →Life satisfaction; Path2: Emotional intelligence → Resilience →Life satisfaction; Path3: Emotional intelligence → Self-efficacy → Resilience → Life satisfaction.

The comparison was conducted to testify whether there were significant differences in indirect effect paths, the result showed that none of the comparisons were significant, with the Bootstrap’s 95% CI contain 0.

## Discussion

The test results indicated that emotional intelligence positively affected the life satisfaction of nurses, which confirmed Hypothesis 1. Diener has pointed out that personality traits are very important to a person’s overall quality and often determine his/her life satisfaction (Diener, 1985). Emotional intelligence thought to be the power to process emotional problems, is strongly positively correlated with life satisfaction in different populations (Delhom et al., 2017; Kasler et al., 2022; Koca, 2022). Particularly in nursing professionals, emotional intelligence related to nursing work can promote positive organizational outcomes, such as preventing occupational burnout (Molero Jurado et al., 2018) and predicting engagement (Pérez-Fuentes et al., 2018). Emotionally intelligent nurses are usually more satisfied with life because they clearly know better ways to deal with the negative emotions that arise in the environment. Moreover, emotionally intelligent people are more likely to understand the emotions of others; that is, they can perceive better and more often feel satisfied when others have positive feelings toward them. At the same time, emotional intelligence also helps individuals explain the reasons for others’ actions and avoid other people’s dissatisfaction for various reasons; hence, they experience positive emotions more often (Escoda and Alegre, 2016). Emotional intelligence is a variable trait that can be enhanced through training course, just like empathy (Cunico et al., 2012). Developing the necessary strategies to regulate or manage individual emotions, and then emotional skills incorporating structures of emotional intelligence can help them better utilize emotional information, facilitating a high level of life satisfaction (Guasp Coll et al., 2020).

After age, educational level, working years, nursing title, and marital status were controlled, the findings revealed that self-efficacy mediated the relationship between emotional intelligence and life satisfaction (Path 1), supporting Hypothesis 2. The mediating effect accounted for 28.95%. In the prior study on nursing staff, self-efficacy greatly impacted anxiety and sleep disturbances during the COVID-19 pandemic (Simonetti et al., 2021) and even positively impacted all aspects of nurses’ general health (Dadipoor et al., 2021). Researchers found that emotional intelligence, working pressure, self-efficacy, and autonomy were of great significance in improving nurses’ job satisfaction and performance (Hwang and Park, 2022). Interestingly, emotional intelligence and self-efficacy are predictors of perceived stress in nurses facing various stressful situations (Molero Jurado et al., 2019). In fact, some researches confirmed that emotional intelligence can strengthen personal internal resources, which is also extremely important to person’s self-efficacy (Gharetepeh et al., 2015; Saeed and Ahmad, 2020; Chang and Tsai, 2022). Moreover, nurses with high emotional intelligence exhibited positive attitudes and strong self-efficacy, which can mitigate the effects of stress on burnout (Yao et al., 2018). Meanwhile, self-efficacy could also promote nursing managers to adopt more positive coping styles in high-pressure work environments and effectively improve their quality of work-life (Zhang et al., 2022), as well as life satisfaction (Hajek and König, 2019).

After age, educational level, working years, nursing title, and marital status were controlled, the results of this study also found that resilience partially mediated between emotional intelligence and life satisfaction (Path 2), with the mediating effect accounted for 23.68%, confirming Hypothesis 3. The more inclined nurses were to emotional intelligence, the stronger their resilience perception ability, which positively impacted their life satisfaction. Resilience, considered an important construct in positive psychology, was significantly correlated with stress, job burnout, and turnover intention of healthcare workers (Eley et al., 2018). Similarly, several factors related to resilience have been seen in research on nursing students, including self-efficacy, optimism, emotional intelligence, and self-care (Hughes et al., 2021). Prior findings have confirmed that the more emotionally intelligent a person is, the more resilient they are to effectively deal with adversity and challenges of high workloads and expectations (Sarrionandia et al., 2018; Chen, 2019). Additionally, resilience has been verified to reduce job burnout, regulate the overall health of nurses (García-Izquierdo et al., 2018), and directly affect the well-being and life satisfaction of nurses (Kim et al., 2019).

After age, educational level, working years, nursing title, and marital status were controlled, it was found that emotional intelligence and life satisfaction were continuously mediated by self-efficacy and resilience (Path 3), with the mediating effect accounted for 17.37%, confirming Hypothesis 4. Self-efficacy and resilience jointly promoted life satisfaction, with high emotional intelligence scores indicating that nurses can reduce the intensity and presence of negative emotions, promote positive coping styles, and maintain an optimistic attitude, which positively impacted the management of stressful situations and the prevention of burnout (Molero Jurado et al., 2019; Lu et al., 2022). Along this line, those nursing professionals with high emotional intelligence gradually formed a perception of competence, namely self-efficacy. As a positive psychological driver, self-efficacy helped individuals regulate their thinking processes, emotions, and behaviors, high levels of self-efficacy were related to better mental health and improved recovery from work stress and adversity (Hu et al., 2020). Self-efficacy enhanced the confidence of nurses to complete nursing tasks and the determination to achieve clinical goals. The self-efficacy of nurses also helped them cultivate and improve their resilience by viewing failures as challenges to learn instead of a threat to avoid (Yu et al., 2019). Personal resilience played a key protective role in dealing with negative situations and emotional disturbance caused by various workplace pressures (Bhattarai et al., 2021). At the same time, interventions to enhance resilience have been proven to reduce the mental burden of nurses and have positive effects on nurses’ well-being and life satisfaction (Janzarik et al., 2022). As nurses become more resilient to stressful work environments, their mental health improves, resulting in increased well-being and life satisfaction.

## Conclusion

The survey indicated that emotional intelligence positively predicted the life satisfaction of Chinese nurses. In addition, it turned out that self-efficacy and resilience played a continuous intermediary effect between emotional intelligence and life satisfaction. According to the study, the influence of emotional intelligence, self-efficacy, and resilience on life satisfaction of Chinese nurses should not be ignored. On the one hand, nurses can participate in some training to strengthen the ability of emotional intelligence, so as to improve their sense of life satisfaction. On the other hand, nursing managers should provide nurses with a favorable working environment from the perspective of positive psychology, improve their sense of self-efficacy, enhance the capacity to cope with challenges and recover from stress, and ultimately improve their life satisfaction.

### Limitations

Although we have demonstrated that self-efficacy and resilience played a continuous intermediary effect between emotional intelligence and life satisfaction, our study still exists some limitations. First, the possible impact of the biases generated by online self-reporting on the accuracy of the data should not be ignored. Second, since cross-sectional data cannot be correlated causally, no causal conclusions can be drawn. Therefore, longitudinal researches or experiments can be added to further verify the results of our study. Finally, this study only involved nurses from various hospitals in China; thus, the findings may not be generalizable to the global nursing profession.

## Data availability statement

The original contributions presented in the study are included in the article/Supplementary material, further inquiries can be directed to the corresponding author.

## Ethics statement

The studies involving human participants were reviewed and approved by the Ethics Committee of Chengdu Fourth Hospital. The patients/participants provided their written informed consent to participate in this study.

## Author contributions

YQ and JL was involved in all aspects of the study and preparation of the manuscript. DW was involved with the design of the study and preparation of the manuscript. All authors contributed to the article and approved the submitted version.

## Funding

This work was supported by the National Natural Science Foundation of China (grant no. 82001444).

## Conflict of interest

The authors declare that the research was conducted in the absence of any commercial or financial relationships that could be construed as a potential conflict of interest.

## Publisher’s note

All claims expressed in this article are solely those of the authors and do not necessarily represent those of their affiliated organizations, or those of the publisher, the editors and the reviewers. Any product that may be evaluated in this article, or claim that may be made by its manufacturer, is not guaranteed or endorsed by the publisher.
